# Fermented Pistachio-Based Beverages as Novel Functional Foods: Improved Phenolic Bioaccessibility and Antidiabetic Properties

**DOI:** 10.3390/foods15101639

**Published:** 2026-05-08

**Authors:** Antonela Guadalupe Garzón, Franco Van de Velde, Maria Cecilia Puppo, Tiziana Di Renzo, Anna Reale, Silvina Rosa Drago

**Affiliations:** 1 Instituto de Tecnología de Alimentos, Facultad de Ingeniería Química, Universidad Nacional del Litoral, Santiago del Estero 2829, Santa Fe 3000, Argentina; an.garzon@hotmail.com.ar (A.G.G.);; 2 Consejo Nacional de Investigaciones Científicas y Técnicas (CONICET), Colectora Ruta Nac. Nº 168, Km. 0, Paraje El Pozo, Santa Fe 3000, Argentina; 3 CIDCA-UNLP-CONICET, 47 y 116 s/n, La Plata 1900, Argentina; mcpuppo@quimica.unlp.edu.ar; 4 Institute of Food Sciences, National Research Council (ISA-CNR), Via Roma 64, 83100 Avellino, Italy; tiziana.direnzo@isa.cnr.it

**Keywords:** pistachio, LAB fermentation, phenolics, bioaccessibility, bioactive properties

## Abstract

This study evaluated the effects of lactic acid bacteria (LAB) fermentation on the phenolic composition, bioaccessibility, and bioactive potential of pistachio-based beverages formulated from the Argentine (A) ‘Kerman’ variety and the Italian (I) ‘Bronte’ variety of pistachios. Beverages were fermented using either *Leuconostoc pseudomesenteroides* PD4 or *Companilactobacillus alimentarius* PG3, producing four fermented samples (APD4, APG3, IPD4, and IPG3), and two non-fermented controls (AC, IC). Fermentation markedly increased the total phenolic content (1.3–1.4-fold) and proanthocyanidins (up to 1.6-fold in APG3). Intestinal and colonic dialysates contained only quercetin, with intestinal bioaccessibility 27.6 times higher in APG3 than in AC, and colonic bioaccessibility 2.4-fold higher. Antioxidant activity (ABTS•+ assay) increased by 5–15% in the fermented samples, whereas FRAP remained stable. While α-amylase inhibition decreased slightly after fermentation, α-glucosidase inhibition reached up to 82% (IPG3), and DPP-IV inhibition peaked at 95% (IPD4). ACE-I inhibition remained high (84–93%). LAB fermentation effectively enhanced the biofunctional properties of pistachio-based beverages, primarily through improved polyphenol bioaccessibility and antidiabetic potential. These findings highlight lactic acid fermentation as a promising biotechnological strategy for developing novel, plant-based functional beverages with enhanced health-promoting properties.

## 1. Introduction

Pistachio nuts (*Pistacia vera* L.) are primarily consumed as snacks but are also widely used as an ingredient in confectionery, fermented meats, ice cream, baked goods, and other food products [1]. Pistachio nuts are considered a nutritious food, providing proteins, dietary fiber, and healthy fats, especially oleic and linoleic acids, along with essential minerals (potassium, magnesium, phosphorus), vitamins (notably vitamin B6 and thiamine), and bioactive compounds such as carotenoids and phenolic compounds [2]. Phenolic acids, some flavonoids (e.g., quercetin derivatives), and condensed tannins or proanthocyanidins, have been identified in the hulls and kernels of pistachio seeds [3,4].

Pistachio nuts are considered part of the Mediterranean diet and their bioactive compounds have been associated with reduced oxidative stress and inflammation. This supports cardiovascular health and may reduce the development of non-communicable diseases such as type II diabetes and certain cancers [5].

Recent developments have introduced pistachio-based beverages as promising substrates for fermentation with lactic acid bacteria (LAB) [6,7,8]. These beverages have demonstrated excellent potential in supporting the growth and survival of LAB, reaching more than 9 log CFU/mL after 24 h of fermentation and maintaining viability for up to 30 days at 4 °C. Thus, these plant-based fermented beverages could represent an innovative alternative for incorporating probiotic bacteria, which are traditionally delivered through dairy products. They are particularly suitable for individuals with lactose intolerance or those following special diets, such as veganism [9,10]. In this context, LAB fermentation can modify the plant matrix, facilitating the release of compounds such as phenolics that are often bound to proteins or fiber carbohydrate constituents. This process enhances their extractability and, consequently, may improve the bioaccessibility and bioavailability of phenolic compounds [11,12]. However, certain LAB strains can utilize phenolic compounds as prebiotics, which may influence their concentration. They can also produce secondary metabolites with distinct bioactive properties or bioaccessibility [13]. On the other hand, LAB can hydrolyze proteins during fermentation, releasing numerous bioactive peptides, which contribute to the enhanced biofunctional potential of the final fermented product [14,15].

To date, no studies have assessed the effects of LAB fermentation on the phenolic compounds and peptide profiles of pistachio-based beverages, nor the resulting impact on their bioaccessibility and bioactive properties following digestion.

Therefore, this study evaluates the effects of fermentation using *Leuconostoc pseudomesenteroides* PD4 or *Companilactobacillus alimentarius* PG3 on beverages made from two varieties of pistachio cultivated in Argentina and Italy. Specifically, changes in phenolic and peptide content and their associated antioxidant, antihypertensive, and antidiabetogenic activities were assessed following simulated gastrointestinal and colonic digestion.

## 2. Materials and Methods

### 2.1. Raw Materials

Pistachio samples used in the study consisted of Pistacia vera L. cv. Bronte (Italy) and cv. Kerman (Argentina), selected as a representative and commercially relevant cultivars in their respective producing regions. Cv. Bronte is a traditional Italian cultivar with Protected Designation of Origin (PDO) status, whereas cv. Kerman is one of the most widely cultivated pistachio varieties worldwide due to its agronomic adaptability and high yield.

The Argentine pistachios (A) of the ‘Kerman’ variety were cultivated in San Juan, Argentina, harvested in 2019, and donated by Pistacho de los Andes (San Juan, Argentina). The Italian pistachios (I) of the ‘Bronte’ variety were also harvested in 2019 and were purchased from the Aroma Sicilia farm in Sicily, southern Italy. The composition of the Argentine and Italian pistachios, expressed as g/100 g of dry weight (dw), was as follows: 27.1 ± 0.2 and 25.7 ± 0.1 for protein, 45.4 ± 1.8 and 48.5 ± 1.7 for crude fat, 10.7 ± 0.8 and 15.3 ± 1.0 for total dietary fiber, 5.2 ± 0.0 and 4.1 ± 0.2 for moisture, and 2.9 ± 0.0 and 3.1 ± 0.1 for ash, respectively.

### 2.2. Pistachio Beverage Preparation

The pistachio beverages were obtained using a colloidal mill (Homomaster 120, S.A.R. Group, Varese, Italy), as previously described [7]. Briefly, 1 kg of pistachios was processed with water at a ratio of 1:5 (pistachios/water) for 5 min under recirculation. The resulting beverages were then heat-treated at 70 °C for 30 min and cooled to 4 °C. Aliquots were fermented with *Leuconostoc pseudomesenteroides* PD4 or *Companilactobacillus alimentarius* PG3 strains, at a final concentration of 6 log colony-forming units (CFU)/mL. The lactic acid bacteria (LAB) used belong to the microbial culture collection of the Institute of Food Sciences, National Research Council (ISA-CNR, Avellino, Italy). Fermentation was carried out at 30 °C for 24 h. Samples were labeled APD4, APG3, IPD4, and IPG3 for Argentine (A) or Italian (I) pistachio beverages fermented with *L. pseudomesenteroides* PD4 (PD4) or *C. alimentarius* PG3 (PG3). Unfermented beverages were used as controls: AC (Argentine control) and IC (Italian control). Lactic acid bacteria count after 24 h of fermentation was <1 log CFU/g(AC), 8.9 ± 0.4 (APD4) log CFU/g, and 8.4 ± 0.6 log CFU/g (APG3); and <1 log CFU/g (IP), 8.8 ± 0.4 log CFU/g (IPD4), and 8.7 ± 0.5 log CFU/g (IPG3).

The median composition of the Argentine and Italian pistachio-based beverages, expressed in g/100 mL, was as follows: 5.17 ± 0.17 and 4.69 ± 0.65 for protein, 8.47 ± 0.40 and 8.16 ± 0.74 for crude fat, 2.01 ± 0.09 and 2.63 ± 0.20 for total dietary fiber, 18.30 ± 0.20 and 17.20 ± 0.09 for total solids, and 0.50 ± 0.03 and 0.50 ± 0.02 for ash, respectively. All samples were lyophilized at −50 °C, 1 mBar for 24 h (Labconco, FreeZone 4.5 L, Kansas City, MO, USA) and stored at −20 °C for further analysis.

### 2.3. Phenolic Compound Analysis

#### 2.3.1. Extract Preparation

Free phenolic compounds were extracted from the pistachio flour and the lyophilized beverages according to the procedure of Garzón et al. [16] with slight modifications. Briefly, 5 mL of 80:20 methanol:water was added to 0.2 g of defatted samples and the mixture was sonicated for 20 min (Arcano, Ningbo, China). The mixture was then centrifuged at 5000 rpm for 20 min using a Cavour VT 3216 centrifuge (Buenos Aires, Argentina), and the resulting supernatants were collected into 15 mL centrifuge tubes. The pellet extractions were repeated using 5 mL of extraction solvent. After centrifugation, the two resulting supernatants were combined and subjected to acid hydrolysis using 2 N HCl at 90 °C for 50 min. The resulting pellets were stored and used for the extraction of bound phenolic compounds. Pellets were treated with 5 mL of 2 N NaOH for 3 h, extracted twice with ethyl acetate, and then the solvent was evaporated. The resulting extract was resuspended in methanol and stored for analysis. All extractions were made in triplicate.

#### 2.3.2. Analysis of Phenolic Compounds by HPLC

Free and bound phenolic compounds were separated using a 100 mm × 3.0 mm, 2.7 μm particle size Poroshell 120 column (Agilent, Santa Clara, CA, USA), using an LC-40B XR (Shimadzu Co., Kyoto, Japan) high-performance liquid chromatograph equipped with a diode array detector (DAD) (SPD M40). Data processing and control was performed using Lab Solutions CS software (v6.81, Shimadzu Co., Kyoto, Japan). Extracts were filtered through a Millipore filter with a pore size of 0.45 μm and separated at a flow rate of 0.4 mL/min and a temperature of 35 °C, using a mobile phase gradient of 0.1% formic acid (A) and acetonitrile (B) at 5–6% B for 2 min, 6–7% B for 4 min, 7% B for 7 min, 7–9% B for 9 min, 9% B for 12 min, 9–12% B for 16 min, 12–14% B for 17 min, 14–16% B for 18 min, 16–18% B for 22 min, 18–25% B for 28 min, 25–28% B for 30 min, 28–30% B for 38 min, 30–100% B for 40 min, 100% B for 45 min, 100–5% B for 47 min, and 5% B for 52 min. Peak identification was performed by comparison of retention times and spectral characteristics (absorbance vs. wavelength) with those of standard compounds. Quantification was made through external standard calibration curves of catechin and epicatechin (4–200 mg/L), rutin and myricetin (1.5–60 mg/L), quercetin (2–40 mg/L), and delphinidin and cyanidin (6.25–50 mg/L). Results were expressed as mg of phenolic compound per 100 g of dry weight (dw). These phenolic compounds (catechin, epicatechin, rutin, myricetin, and quercetin) were selected based on their known occurrence in pistachio nuts [17,18,19], and their presence may also be influenced by fermentation processes, which can modify the phenolic profile and enhance the release of bound forms.

#### 2.3.3. Estimation of Total Proanthocyanidins by Acid Hydrolysis

Total proanthocyanidin (PAC) content was estimated following a patented method [20]. Briefly, delphinidin and cyanidin were determined in the acid-hydrolyzed samples obtained for free phenolic compound analysis by HPLC. During hydrolysis, PACs are cleaved to release anthocyanidins. According to the established model, one proanthocyanidin polymer with a degree of polymerization ‘n’ yields ‘n/2’ molecules of anthocyanidins, regardless of the value of ‘n’. To estimate the total PAC content, the amount of anthocyanidins (delphinidin plus cyanidin) was multiplied by 2. The results were expressed as mg PAC/100 g dw.

#### 2.3.4. Estimation of Total Proanthocyanidins by DMAC Method

The 4-(dimethylamino)-cinnamaldehyde (DMAC) method was employed to analyze the content of PACs in samples [21]. PAC content was calculated using an external calibration curve of catechin (1.56–50 mg/L), and results were expressed as mg of catechin equivalents per 100 g of dry weight.

#### 2.3.5. Estimation of Total Phenolic Compounds

The content of total phenolic compounds was calculated as the sum of free, PACs, and bound phenolic compounds detected by HPLC.

### 2.4. Simulated Gastrointestinal Digestion with Colonic Fermentation

The in vitro method described by Van de Velde et al. [22] was applied to simulate the gastrointestinal digestion and colonic fermentation of beverages. For the gastric phase, 1 g of lyophilized samples was rehydrated with 10 mL of water, pH was adjusted to 2, and 0.4 mL of 16 g pepsin/100 mL (P-7000, Sigma-Aldrich, St. Louis, MO, USA, prepared in HCl 0.1 mol/L, with a pepsin activity of 2000 U/mL) was added. The mixtures were incubated in a shaking bath (Dubnoff, Buenos Aires, Argentina) at 37 °C for 2 h. At the end of the pepsin digestion stage, dialysis bags (Spectra/Pore^®^ I dialysis tubing, cut-off 6000 to 8000 Da, Fisher Scientific, Singapore) containing 10 mL NaHCO_3_ buffer were placed in each beaker and incubated for 50 min in a shaking water bath at 37 °C. NaHCO_3_ buffer molarity was calculated to obtain a final pH of 7.0 ± 0.2 in the digest-dialysate. Then, 0.4% pancreatin (P-1750, Sigma-Aldrich) was added to each beaker, and the incubation continued for a further 2 h (pancreatin activity: 3.64 U mL/L). After this process, bag contents corresponding to the “dialysates of the intestinal phase” were transferred to tared flasks, weighed, and frozen at −20 °C until analysis.

To simulate the colonic fermentation phase, the cecal content from male Wistar rats was used as an inoculum at a concentration of 100 g/L. Aliquots of the intestinal digests were mixed with 8 mL of fermentation medium (sterile anaerobic medium with thioglycolate as an indicator, Laboratorios Britania S.A., Buenos Aires, Argentina) and 2 mL of inoculum. Then, dialysis bags containing 10 mL of fermentation medium were placed in each beaker. Finally, fermentation beakers were placed in a GasPak™ EZ Anaerobe Container System in an oxygen-free CO_2_-saturated atmosphere, and the system was placed in a shaking oven at 37 °C for 24 h. Controls were included in the experiment: 6 g/100 mL of raffinose as a completely fermentable substrate. Blanks were prepared by replacing the sample with water and were subjected to the same digestive process. After incubation, the fermentation process was stopped by adding 2.5 mL of 1 mol/L NaOH. Bag contents corresponding to “dialysates of the colonic fermentation phase” were weighed and frozen at −20 °C until analysis. Analyses were conducted in duplicate.

#### Peptide Analysis by FPLC

Fast peptide liquid chromatography (FPLC) of the obtained intestinal dialysates was performed according to Aquino et al. [23]. Fractionation was performed using a KNAUER AZURA system (Berlin, Germany), which was equipped with a Superdex 10/300 GL column (GE Life Sciences, Piscataway, NJ, USA). The molecular mass of the peptide fractions was estimated by comparison with molecular weight standards ranging from 7 kDa to 100 Da. In addition, the relative peak area for each peak was calculated as the peak area divided by the total chromatographic area.

### 2.5. Analysis of Phenolic Compounds and Bioactive Properties of Dialysates and Digests

#### 2.5.1. Analysis of Phenolic Compounds and Estimation of Bioaccessibility

Phenolic compounds in dialysates and digests were analyzed by HPLC, as detailed in Section 2.3.2. Intestinal bioaccessibility (IB%) and colonic bioaccessibility (CB%) were calculated as the percentage of each dialyzable phenolic compound relative to its content in the undigested sample.

#### 2.5.2. Analysis of Antioxidant Capacity

The antioxidant activity (AOA) was assessed by measuring the inhibition of 2, 2′-azinobis (3-ethylbenzothiazoline-6-sulfonic acid) (ABTS•+) according to Re et al. [24]. Ferric reducing antioxidant power (FRAP) was determined according to Benzie et al. (1996) [25]. FRAP and ABTS•+ results were expressed as mmol/L Trolox.

#### 2.5.3. Anti-Diabetogenic Activity

Anti-diabetogenic activity (ADA) was determined by evaluating the inhibition of dipeptidyl peptidase IV (DPP-IV) in intestinal dialysates [26], and the inhibition of α-amylase and α-glucosidase in intestinal digests [27]. Inhibitory effects were expressed as inhibition percentage (%).

#### 2.5.4. Antihypertensive Activity

The antihypertensive activity was analyzed through the angiotensin-converting enzyme (ACE-I) inhibitory activity by the intestinal dialysates, determined according to Hayakari et al. [28]. Inhibitory effects were expressed as inhibition percentage (%).

### 2.6. Statistical Analysis

All results were expressed as mean ± standard deviation (SD). Statgraphics Centurion XV 15.2.06 software was used to analyze the data by analysis of variance (one-way ANOVA). Duncan’s multiple range tests were used to determine differences between samples (*p* < 0.05).

## 3. Results and Discussion

### 3.1. Phenolic Compounds in Pistachio Nuts from Argentina and Italy

Table 1 shows the phenolic compounds extracted from pistachios from Argentina (A, ‘Kerman’ variety) and Italy (I, ‘Bronte’ variety). As shown, flavonoids were recovered in free (F) and/or bound (B) forms. (+)-Catechin and (-)-epicatechin were detected exclusively in bound form in both A and I samples, and were released only after alkaline hydrolysis. Both flavan-3-ol concentrations were more than 10 times higher in I samples compared to A samples. (+)-Catechin, (-)-epicatechin, and other flavan-3-ols are monomeric units of the proanthocyanidins (PACs), also known as condensed tannins [29].

These polyphenolic polymers can be hydrolyzed under hydroalcoholic acidic conditions and high temperatures, converting them into pigmented anthocyanidins. Specifically, PACs composed of (+)-catechin and (−)-epicatechin yield cyanidin, whereas those formed from (+)-gallocatechin and (−)-epigallocatechin produce delphinidin upon depolymerization [30,31]. In this context, both delphinidin and cyanidin were detected in the hydrolyzed extracts from I. In contrast, A extracts only presented cyanidin after acid hydrolysis, revealing differences in the PAC monomer conformation for both pistachio varieties. Results suggest that variety I accumulates PACs based on the four flavan-3-ols, and variety A accumulates PACs based mainly on catechin and epicatechin. PAC content was estimated based on the concentrations of cyanidin and delphinidin formed for both varieties. As can be seen in Table 1, the PAC content from I was more than 7 times higher than that in A. PAC content in the pistachio variety ‘Bronte’ was determined by Gentile et al. [32] using the acid hydrolysis method. The authors reported a value of 268.12 mg of PACs per 100 g of edible nut, a value higher than that obtained in the present study. This discrepancy is likely due to differences in the extraction procedures and hydrolysis conditions.

In the same way, PAC content analyzed by the DMAC method was more than 3 times higher in IP (84.86 ± 4.44 mg catechin equivalents/100 g dw) than in A (27.59 ± 2.0 mg catechin/100 g dw). Similarly, PAC content was determined in the pistachio varieties ‘Bronte’ and ‘Kerman’ by the DMAC method [33]. These authors reported that PACs for both varieties were only present in the pistachio skin, while being absent in the pistachio seed, with PAC contents of 177.57 and 88.51 mg PAC-A2/g dw of skin for ‘Bronte’ and ‘Kerman’ pistachios, respectively. Although these PAC levels were higher than those obtained in the present study, this is likely due to the fact that they were exclusively quantified in the kernel skin. The trend of greater PAC content in ‘Bronte’ compared to ‘Kerman’ aligns with our findings.

The flavonols rutin, myricetin, and quercetin were also detected in extracts from both pistachio varieties. Rutin, in its free form, was found to be 3.3 times higher in extracts A than in I. Myricetin was only found in its free form in the I extracts. Quercetin was recovered in both free and bound forms from the two varieties, and the concentration was slightly higher in I than in A. In agreement with these results, quercetin and myricetin-based flavonols were detected in pistachio kernels by Noguera-Artiaga et al. [34].

The total phenolic content, calculated as the sum of free (excluding anthocyanins) and bound phenolic compounds and PACs, was six times higher in I than in A, primarily due to the higher PAC concentration observed in the Italian variety. Results align with the findings of other investigations that pointed out the ‘Bronte’ variety as a richer phenolic compound source, compared to the ‘Kerman’ variety [33].

The observed qualitative e quantitative differences in phenolic profiles between Argentine and Italian pistachio nuts can be attributed to several factors, including cultivar, geographical origin and environmental conditions (e.g., climate, water availability, and abiotic stress), as well as agronomic practices and harvesting time, as consistently reported by previous studies [35,36,37].

### 3.2. Effect of LAB Fermentation on the Phenolic Content in Pistachio-Based Beverages

The changes in phenolic compounds due to beverage processing and fermentation are presented in Table 2. The unfermented beverages obtained from Argentine (AC) and Italian pistachios (IC) showed a reduction in total phenolic content of up to 70% compared to unprocessed kernels (A and I). This is likely attributable to the degradation of phenolic compounds during beverage processing, particularly during the heat treatment (30 min at 70 °C), which may have promoted the heat-induced oxidation of labile phenolic compounds, as also highlighted by Munekata et al. [38].

Fermenting the Argentine pistachio beverage (AC) with *L. pseudomesenteroides* PD4 (yielding APD4) resulted in only minor changes to the content of phenolic compounds. Fermentation slightly increased the levels of delphinidin, an anthocyanidin absent from both unfermented A and AC, and increased the free form of quercetin. Conversely, rutin and the bound form of quercetin decreased to undetectable levels (Table 2). Fermenting AC with *C. alimentarius* PG3 (resulting in APG3) increased the levels of the anthocyanins delphinidin and cyanidin, resulting in a PAC content increment of 1.6 times compared to AC. Also, APG3 showed no changes in the contents of myricetin and the free form of quercetin (Table 2). Collectively, these changes led to a total phenolic content in APG3 that was 1.3 times higher than that observed in AC. LAB fermentation is a powerful biotechnological strategy to modulate the profile, concentration, and bioactivity of phenolic compounds in food matrices [39]. This process promotes the release of bound phenolics from the food matrix through microbial enzymatic activity (e.g., cellulases, xylanases, and amylases), which degrade cell wall structures, thereby enhancing the solubility, bioaccessibility, and bioavailability of these compounds [40,41]. In addition, microbial metabolism drives key biochemical transformations, such as deglycosylation, ring cleavage, decarboxylation, and oxidation, leading to the formation of simpler, often more bioactive, phenolic derivatives [42,43]. Thus, the overall phenolic content after fermentation reflects a balance between release and degradation processes. While the liberation of bound phenolics and the synthesis of new metabolites may increase total phenolic content, reductions can occur due to further microbial degradation, interactions with other macromolecules, or strain-specific metabolic pathways.

Similarly, fermentation induced significant changes in the phenolic composition of IPD4, with increases in delphinidin, cyanidin, proanthocyanidins (PACs), myricetin, and quercetin, alongside decreases in catechin and rutin. These modifications resulted in a 1.4-fold increase in the total phenolic content compared to the unfermented control (IC). In the same way, IPG3 also exhibited 1.3 times increase in the total phenolic compound content compared to IC (Table 2). PAC changes analyzed by the DMAC method showed the same tendency as that observed using the acid hydrolysis method.

### 3.3. Effects of In Vitro Gastrointestinal Digestion with Colonic Fermentation on Phenolic Compound Bioaccessibility and Peptides in Fermented Pistachio-Based Beverages

All the prepared beverages were subjected to an in vitro gastrointestinal digestion process that included colonic fermentation. Quercetin was the only native pistachio phenolic compound identified in both the intestinal and colonic dialysates. Table 3 presents the intestinal (IB%) and colonic (CB%) bioaccessibility of quercetin in unfermented and fermented beverages from both pistachio varieties. Fermentation with PD4 and PG3 strains increased the IB% of quercetin in beverages from both pistachio varieties.

Quercetin IB% for APG3 was 27.6 times higher than that observed for AC. On the other hand, quercetin was detected only in the colonic dialysates of AC, APD4, APG3, and IPD4 (see Table 3). Notably, the CB% of quercetin in APG3 was 2.4 times higher than that of AC. LAB produces enzymes such as β-glucosidase that can release phenolic compounds bound to fibers or proteins within the food matrix, thereby enhancing their digestive extractability and subsequent bioaccessibility [44]. Although the total quercetin content in APG3 remained similar to that of AC (Table 2), fermentation enhances its intestinal bioaccessibility. Evidence suggests that, although LAB can metabolize phenolic compounds and utilize them as prebiotic substrates, the structural modifications induced in the food matrix during fermentation may enhance the release and extractability of these compounds during digestion, thereby increasing their bioaccessibility [40,45]. At the same time, the colonic microbiota can release and metabolize phenolic compounds into bioactive metabolites [46]. Although these metabolites were not identified or quantified in the present study, they deserve further exploration.

**Figure 1:**
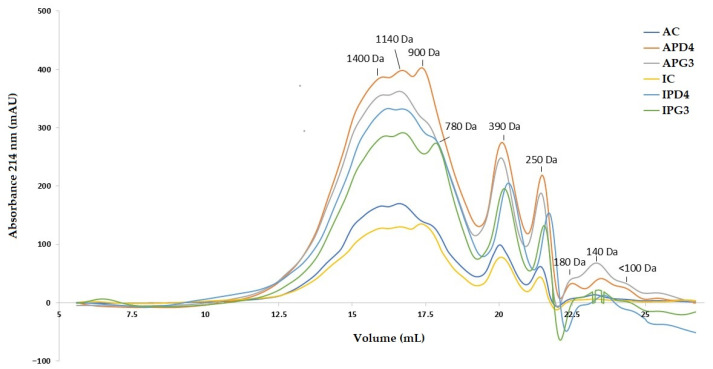
FPLC gel filtration profiles of the dialysates obtained after the in vitro gastrointestinal digestion of pistachio-based beverages.

The FPLC chromatograms of the dialysates from pistachio-based beverages revealed distinct peptide distribution patterns depending on both pistachio variety and fermentation treatment (Figure 1). The separation profile showed a major peptide population eluting between approximately 15 and 18 mL, corresponding to molecular weights in the range of ~1400 to 900 Da, followed by secondary peaks at lower molecular weights (~780, 390, and 250 Da), and minor fractions below 200 Da. For the Argentine variety, the control sample (AC) exhibited the lowest overall signal intensity across the chromatogram, particularly in the main peptide region (1400–900 Da), indicating a lower abundance of soluble peptides after digestion. In contrast, both fermented samples (APD4 and APG3) showed a marked increase in absorbance in this region, with APD4 presenting the highest peak intensities, especially around ~1140 and 900 Da. A similar trend was observed for the Italian variety. The control sample (IC) displayed relatively low signal intensity throughout the profile, whereas fermentation (IPD4 and IPG3) significantly increased peptide abundance. Among these, IPD4 showed a more pronounced accumulation of peptides in the 900–1400 Da range, while IPG3 exhibited a comparatively broader distribution extending towards lower molecular weights. These results suggest that fermentation enhanced the release of peptides that could be bioaccessible after gastrointestinal digestion. As is well established, LAB metabolize proteins and oligopeptides via a coordinated proteolytic system comprising extracellular protein degradation, peptide transport, intracellular peptide hydrolysis, and subsequent amino acid catabolism [47].

### 3.4. Bioactivity of Fermented Pistachio-Based Beverages After In Vitro Gastrointestinal Digestion

**Figure 2:**
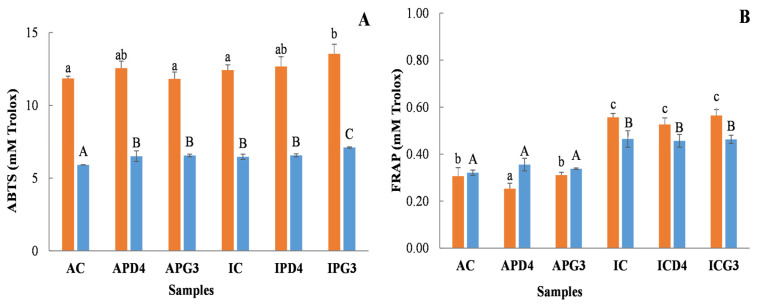
Antioxidant capacity of intestinal (orange bars) and colonic (blue bars) digests from pistachio-based beverages fermented with *Leuc. pseudomesenteroides* PD4 or *C. alimentarius* PG3, yielding APD4, APG3 (Argentine beverages fermented with PD4 and PG3, respectively) and IPD4 and IPG3 (Italian beverages fermented with PD4 and PG3, respectively). Non-fermented beverages served as controls (AC, IC). Analyzed by the ABTS^+^ inhibition method (**A**); analyzed by the FRAP method (**B**). Different letters above the bars indicate statistically significant differences among samples (lowercase letters indicate differences between intestinal samples; uppercase letters indicate differences between colonic samples). Statistical analysis was performed using one-way ANOVA followed by Duncan’s multiple range tests, with a significance level of *p* < 0.05.

Figure 2A presents the antioxidant capacity of the intestinal and colonic digests of the beverages, as measured by the ABTS^+^ assay. Fermentation led to a slight increase in the antioxidant capacity of IPG3 intestinal digest compared to IC. A similar trend was observed in the colonic digests, except for IPD4, which did not show a significant increase.

This enhancement is likely due to improved phenolic compound extractability during fermentation and the generation of bioactive metabolites by the colonic microbiota. Both of these factors may contribute to the increased antioxidant capacity observed in the digested samples. Therefore, these improvements may translate into localized antioxidant effects in the gastrointestinal tract [48]. In contrast, the antioxidant capacity of the intestinal and colonic digests analyzed with the FRAP method did not increase as a result of LAB fermentation (Figure 2B). However, a marked difference was observed between pistachio varieties, with higher FRAP values in digests derived from the Italian variety, reflecting their greater phenolic content (Table 1 and Table 2). FRAP and ABTS^+^ assays evaluate antioxidant capacity through different reaction mechanisms. The FRAP method is based solely on electron transfer (ET), measuring the ability of antioxidants to reduce ferric (Fe^3+^) to ferrous (Fe^2+^) ions. In contrast, the ABTS^+^ assay involves both ET and hydrogen atom transfer mechanisms (HAT), allowing it to detect a broader range of antioxidant compounds [49]. These mechanistic differences can explain the changes in antioxidant capacity for the digested samples. Samples likely contain phenolic compounds and, potentially, bioactive peptides (which were not evaluated in this study) that contribute to antioxidant activity through both ET and HAT mechanisms, thereby resulting in higher antioxidant capacity values in the ABTS assay compared to FRAP.

**Figure 3:**
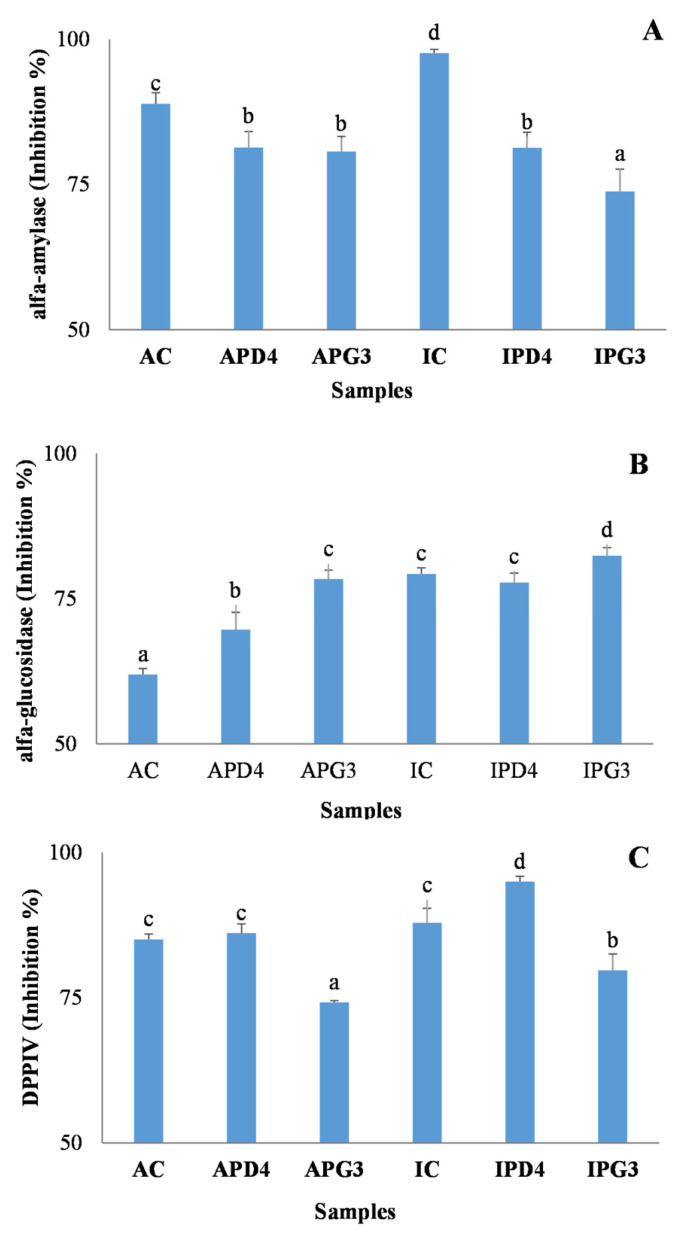
Inhibition of α-amylase (**A**) and α-glucosidase (**B**) of digests, and DPP-IV of dialysates (**C**) from pistachio-based beverages fermented with *Leuc. pseudomesenteroides* PD4 or *C. alimentarius* PG3, yielding APD4, APG3 (Argentine) and IPD4, IPG3 (Italian), respectively. Non-fermented beverages served as controls (AC, IC). Different letters indicate differences between samples (*p* < 0.05).

Figure 3 shows the antidiabetogenic activity of the intestinal digests of pistachio-based beverages. As shown in Figure 3A, all samples exhibited strong α-amylase inhibitory activity, with inhibition reaching up to 95%. The unfermented controls (IC and AC) demonstrated the highest inhibitory results, while the fermented beverages showed a slightly lower, yet still significant, inhibition. This strong α-amylase inhibition is likely due to the high content of PACs in pistachios, which may form enzyme–tannin complexes, avoiding the interaction between α-amylase and its starch substrates [50]. Although fermentation enhanced the extractability of proanthocyanidins (PACs) (Table 2), it may also have altered the polymer structure, thereby reducing their ability to precipitate proteins and inhibit the α-amylase.

In contrast to α-amylase, fermentation seems to increase the α-glucosidase inhibitory activity, particularly for the Argentine variety. Figure 3B presents the effects of intestinal digests on the α-glucosidase inhibition. Digested samples exhibited enzyme inhibition ranging from 61% to 82%, with IPG3 presenting the highest inhibitory activity. This improvement is likely attributed to the increased availability of flavonoids, such as quercetin, which are known potent inhibitors of α-glucosidase [51]. Lalegani et al. [52] reported that the α-glucosidase inhibitory activity of pistachio extracts was primarily attributed to their non-tannin phenolic fraction. As previously discussed, the fermentation of beverages enhanced the extractability of phenolic compounds, facilitating the release of flavonoids as quercetin during digestion. These flavonoids may then exert a greater inhibitory effect on α-glucosidase that is observed in non-fermented samples. As seen in Table S1, rutin and quercetin flavonols were recovered at higher concentrations in digests from fermented beverages than in their respective controls, for both pistachio varieties.

However, IC displayed higher α-glucosidase inhibition values than APD4 and APG3, despite its lower flavonol content. This suggests that the inhibitory effect cannot be attributed solely to the analyzed flavonols and may involve other compounds, such as bioactive peptides, as well as potential synergistic interactions among different constituents.

The inhibition of DPP-IV was evaluated in the intestinal dialysates (Figure 3C). The dialysate from IPD4 exhibited the highest inhibitory activity, reaching approximately 95%, slightly higher than that of the unfermented beverage IC (86%). In contrast, fermentation with PG3 appeared to reduce the DPP-IV inhibitory activity of the intestinal dialysates for both pistachio varieties, likely due to strain-dependent alterations affecting the enzyme-inhibitory properties of the samples. Conversely, fermentation with PD4 seemed to maintain or even enhance the DPP-IV inhibition capacity. Inhibiting DPP-IV is beneficial for glycemic control, as it prolongs the activity of incretin hormones such as GLP-1 and GIP, thereby promoting postprandial insulin secretion. Consequently, various flavonoids and other phenolic compounds have been proposed as natural DPP-IV inhibitors, offering a promising dietary strategy for managing type 2 diabetes [53,54]. In this context, pistachio-based beverages, with or without fermentation by PD4, may serve as a promising source of natural DPP-IV inhibitors, potentially supporting glucose regulation.

**Figure 4:**
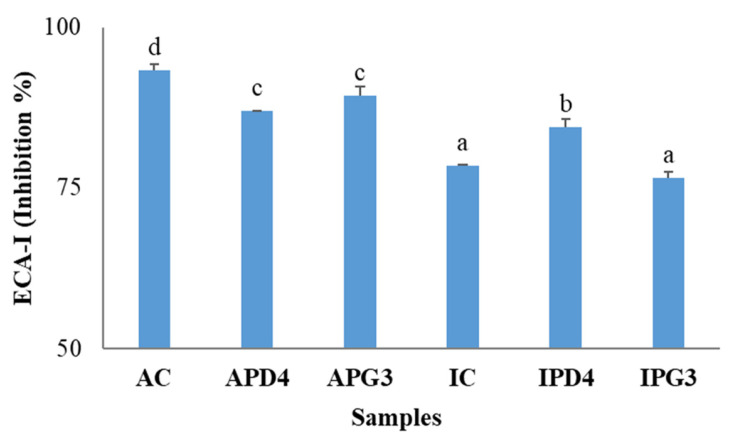
Inhibition of ACE-1 of dialysates from pistachio beverages fermented with *Leuc. pseudomesenteroides* PD4 or *C. alimentarius* PG3, yielding APD4, APG3 (Argentine) and IPD4, IPG3 (Italian), respectively. Non-fermented beverages served as controls (AC, IC). Different letters indicate differences between samples (*p* < 0.05).

Lastly, the antihypertensive potential of the beverage dialysates was evaluated through ACE-I inhibition. Intestinal dialysates from Argentine pistachio-based beverages exhibited higher ACE-I inhibition compared to those made from the Italian nuts. Among all samples, the dialysate from AC exhibited the highest ACE-I inhibitory activity (93.2%). However, fermentation of the Argentine pistachio beverage slightly reduced the enzyme inhibition capacity to 87.0–89.4% (Figure 4). Contrarily, fermentation of the Italian pistachio beverage with PD4 resulted in a slight but significant increase in ACE-I inhibition (enzyme inhibition of 84.5% by IPD4 vs. 78.5% by IC).

Phenolic compounds can inhibit ACE-I by interacting with its active site, contributing to potential blood pressure regulation [55]. However, bioactive peptides produced during fermentation or gastrointestinal digestion have been demonstrated to have stronger inhibitory effects on ACE-I [56]. In this regard, as seen in Figure 1, fermented beverages showed a clear shift toward lower molecular weight peptides (mainly <1.5 kDa), compared to their respective controls. This is particularly relevant, as peptides within this size range have been widely associated with enhanced ACE-I inhibitory effects, as well as improved intestinal absorption [57,58]. The FPLC results support the hypothesis that LAB fermentation not only improves phenolic bioaccessibility but also promotes the generation of low-molecular-weight peptides during digestion, which may significantly contribute to the observed ECA-I inhibition, at least for IPD4. These findings suggest that ACE-I inhibitory activity cannot be attributed exclusively to either phenolic compounds or peptides. Instead, both fractions may act together, potentially through additive or synergistic mechanisms.

## 4. Conclusions

This study highlights the potential of lactic acid bacteria fermentation to enhance the functional quality of pistachio-based beverages. Fermentation increased total phenolic content by 1.3–1.4-fold in APG3, IPD4, and IPG3 compared to their unfermented counterparts. Quercetin intestinal bioaccessibility was 27.6 times higher in APG3 than in AC, and its colonic bioaccessibility was also enhanced (2.4-fold vs. AC). Fermentation improved antioxidant activity as measured by the ABTS^+^ assay (5–15% increase), while FRAP values remained unchanged, reflecting the assay-dependent nature of antioxidant measurement. Regarding antidiabetic potential, α-amylase inhibition was highest for the AC (around 95%) but slightly decreased after fermentation, likely associated with structural modifications of proanthocyanidins during fermentation. In contrast, α-glucosidase inhibition increased during fermentation, being the highest in IPG3 (82%). DPP-IV inhibition reached 95% in IPD4, although fermentation with PG3 decreased this activity in both varieties, suggesting strain-specific effects. AC exhibited the highest ACE-I inhibitory activity (93.2%), but its fermentation slightly reduced the enzyme inhibition capacity. Conversely, fermentation of the Italian pistachio beverage with PD4 resulted in a slight but significant increase in ACE-inhibitory activity, likely associated with an increased abundance of low-molecular-weight peptides released during LAB fermentation. Overall, fermentation, especially with the PD4 strain, enhanced bioactivity, supporting pistachio-based fermented beverages as promising functional, plant-based products.

Future studies employing LC-MS/MS-based metabolomic approaches and in silico molecular docking analyses are warranted to further elucidate the specific phenolic compounds responsible for the observed bioactivities and to better clarify their mechanisms of action.

## Figures and Tables

**Table 1:** Content of phenolic compounds in Argentine (A) and Italian (I) pistachio nuts.

Phenolic Compound (mg/100 g dw)		A	I
(+)-Catechin	F	-	-
	B	0.14 ± 0.01 ^a^	1.27 ± 0.21 ^b^
(-)-Epicatechin	F	-	-
	B	0.02 ± 0.00 ^a^	0.25 ± 0.05 ^b^
Delphinidin	F	-	5.55 ± 0.49 ^c^
	B	-	-
Cyanidin	F	5.07 ± 0.22 ^b^	31.58 ± 1.37 ^e^
	B	-	-
Rutin	F	1.44 ± 0.44 ^d^	0.44 ± 0.03 ^bc^
	B	-	-
Myricetin	F	-	0.82 ± 0.05 ^e^
	B	-	-
Quercetin	F	1.19 ± 0.03 ^a^	2.29 ± 0.07 ^d^
	B	0.01 ± 0.00 ^b^	0.01 ± 0.02 ^c^
PACs *		10.14 ± 0.44 ^b^	74.27 ± 3.73 ^e^
Total **		12.93 ± 0.84 ^b^	79.35 ± 3.55 ^e^

* Proanthocyanidins (PACs) are equal to the contents of cyanidin and delphinidin multiplied by 2. ** Total equals free (F) phenolic content (excluding cyanidin and delphinidin, which appeared after acid hydrolysis of free phenolic extracts and are only used for PAC estimation), bound (B) phenolic compounds, and PAC content. Different lowercase letters within the same row indicate significant differences (*p* ≤ 0.05).

**Table 2:** Changes in the phenolic compound content of fermented beverages from Argentine and Italian pistachios.

Phenolic Compounds (mg/100 g dw)		Argentine Beverages			Italian Beverages		
		AC	APD4	APG3	IC	IPD4	IPG3
Catechin	F	-	-	-	-	-	-
	B	0.03 ± 0.01 ^a^	-	-	0.50 ± 0.07 ^a^	0.003 ± 0.000 ^a^	-
Epicatechin	F	-	-	-	-	-	-
	B	0.08 ± 0.01 ^a^	0.05 ± 0.00 ^a^	0.06 ± 0.00 ^a^	0.09 ± 0.01 ^a^	0.09 ± 0.01 ^a^	0.06 ± 0.02 ^a^
Delphinidin	F	-	0.05 ± 0.00 ^a^	0.11 ± 0.02 ^a^	0.35 ± 0.01 ^a^	0.88 ± 0.15 ^b^	1.08 ± 0.12 ^b^
	B	-	-	-	-	-	-
Cyanidin	F	2.51 ± 0.14 ^a^	2.95 ± 0.36 ^a^	3.81 ± 0.20 ^ab^	8.68 ± 0.06 ^c^	12.18 ± 0.80 ^d^	11.37 ± 1.60 ^d^
	B	-	-	-	-	-	-
Rutin	F	1.51 ± 0.24 ^d^	-	0.75 ± 0.03 ^c^	1.43 ± 0.03 ^d^	-	0.30 ± 0.10 ^ab^
	B	-	-	-	-	-	-
Myricetin	F	0.21 ± 0.07 ^bc^	0.11 ± 0.05 ^b^	0.26 ± 0.01 ^c^	0.30 ± 0.01 ^cd^	0.60 ± 0.00 ^f^	0.39 ± 0.09 ^de^
	B	-	-	-	-	-	-
Quercetin	F	1.00 ± 0.14 ^a^	1.59 ± 0.01 ^b^	1.15 ± 0.11 ^a^	2.02 ± 0.05 ^c^	3.67 ± 0.03 ^f^	2.64 ± 0.08 ^e^
	B	0.005 ± 0.000 ^a^	-	-	0.01 ± 0.00 ^b^	0.01 ± 0.00 ^ab^	0.005 ± 0.001 ^ab^
PACs *		5.02 ± 0.27 ^a^	5.99 ± 0.72 ^ab^	7.85 ± 0.43 ^ab^	18.05 ± 0.14 ^c^	26.12 ± 1.91 ^d^	24.90 ± 2.96 ^d^
Total **		7.85 ± 0.72 ^a^	7.75 ± 0.75 ^a^	10.07 ± 0.53 ^b^	22.41 ± 0.24 ^c^	30.50 ± 1.87 ^d^	28.30 ± 2.90 ^d^

* Proanthocyanidins (PACs) are equal to the contents of cyanidin and delphinidin multiplied by 2. ** Total equals free (F) phenolic content (excluding cyanidin and delphinidin, which appeared after acid hydrolysis of free phenolic extracts and are only used for PAC estimation), bound (B) phenolic compounds, and PAC content. Different lowercase letters within the same row indicate significant differences (*p* ≤ 0.05).

**Table 3:** Intestinal and colonic quercetin bioaccessibility from pistachio beverages.

	Quercetin		
Sample	IB %	CB %	TB %
AC	0.05 ± 0.03 ^aA^	0.97 ± 0.26 ^bB^	1.02 ± 0.23 ^bB^
APD4	0.25 ± 0.10 ^abAB^	0.05 ± 0.01 ^aA^	0.31 ± 0.10 ^bA^
APG3	1.38 ± 0.34 ^aC^	2.33 ± 0.37 ^abC^	3.72 ± 0.71 ^bC^
IC	0.22 ± 0.12 ^aA^	-	0.22 ± 0.12 ^aA^
IPD4	0.56 ± 0.11 ^aB^	0.31 ± 0.01 ^aA^	0.87 ± 0.11 ^bAB^
IPG3	0.55 ± 0.34 ^aB^	-	0.55 ± 0.34 ^aAB^

IB: intestinal bioaccessibility, CB: colonic bioaccessibility, TB: total bioaccessibility. Different lowercase letters within the same row indicate significant differences (*p* ≤ 0.05). Different uppercase letters within the same column indicate significant differences (*p* ≤ 0.1).

## Data Availability

The original contributions presented in this study are included in the article/Supplementary Materials. Further inquiries can be directed to the corresponding authors.

## Supplementary Materials

The following supporting information can be downloaded at: https://www.mdpi.com/article/10.3390/foods15101639/s1, Table S1. Phenolic compound content in intestinal digests from Argentine and Italian pistachio-based beverages.

## Abbreviations

The following abbreviations are used in this manuscript:

A	Argentine pistachio sample
ABTS•+	2,2′-azinobis(3-ethylbenzothiazoline-6-sulfonic acid) radical cation
AC	Argentine control (non-fermented beverage)
ACE-I	Angiotensin-converting enzyme I
ADA	Anti-diabetogenic activity
APD4	Argentine pistachio beverage fermented with Leuconostoc pseudomesenteroides PD4
APG3	Argentine pistachio beverage fermented with Companilactobacillus alimentarius PG3
B	Bound phenolic fraction
CB%	Colonic bioaccessibility
CFU	Colony-forming units
DPP-IV	Dipeptidyl peptidase IV
dw	Dry weight
F	Free phenolic fraction
FPLC	Fast protein liquid chromatography
FRAP	Ferric reducing antioxidant power
HPLC	High-performance liquid chromatography
I	Italian pistachio sample
IB%	Intestinal bioaccessibility
IC	Italian control (non-fermented beverage)
IPD4	Italian pistachio beverage fermented with Leuconostoc pseudomesenteroides PD4
IPG3	Italian pistachio beverage fermented with Companilactobacillus alimentarius PG3
LAB	Lactic acid bacteria
PACs	Proanthocyanidins
TB%	Total bioaccessibility

## References

[1] Higgs J., Styles K., Carughi A., Roussell M.A., Bellisle F., Elsner W., Li Z. (2021). Plant-based snacking: Research and practical applications of pistachios for health benefits. J. Nutr. Sci..

[2] Mandalari G., Barreca D., Gervasi T., Roussell M.A., Klein B., Feeney M.J., Carughi A. (2022). Pistachio nuts (*Pistacia vera* L.): Production, nutrients, bioactives and novel health effects. Plants.

[3] Erşan S., Güçlü Üstündaǧ Ö., Carle R., Schweiggert R.M. (2016). Identification of phenolic compounds in red and green pistachio (*Pistacia vera* L.) hulls (exo- and mesocarp) by HPLC-DAD-ESI-(HR)-MSn. J. Agric. Food Chem..

[4] Tomaino A., Martorana M., Arcoraci T., Monteleone D., Giovinazzo C., Saija A. (2010). Antioxidant activity and phenolic profile of Pistachio (*Pistacia vera* L., Variety Bronte) seeds and skins. Biochimie.

[5] Khadivi A., Nikoogoftar-Sedghi M., Tunç Y. (2025). Agronomic characteristics, mineral nutrient content, antioxidant capacity, biochemical composition, and fatty acid profile of iranian pistachio (*Pistacia vera* L.) cultivars. BMC Plant Biol..

[6] Garzón A.G., Puppo M.C., Di Renzo T., Drago S.R., Reale A. (2024). Mineral bioaccessibility of pistachio-based beverages: The effect of lactic acid bacteria fermentation. Plant Foods Hum. Nutr..

[7] Reale A., Puppo M.C., Boscaino F., Garzón A.G., Drago S.R., Marulo S., Di Renzo T. (2024). Development and evaluation of a fermented pistachio-based beverage obtained by colloidal mill. Foods.

[8] Di Renzo T., Osimani A., Marulo S., Cardinali F., Mamone G., Puppo C., Garzón A.G., Drago S.R., Laurino C., Reale A. (2023). Insight into the role of lactic acid bacteria in the development of a novel fermented pistachio (*Pistacia vera* L.) beverage. Food Biosci..

[9] Messadi N., Mechmeche M., Setti K., Tizemmour Z., Hamdi M., Kachouri F. (2023). Consumer perception of a new non-dairy functional beverage optimized made from lactic acid bacteria fermented date fruit extract. Int. J. Gastron. Food Sci..

[10] Di Renzo T., Reale A., Nazzaro S., Marena P., Rahim M.H.A., Mohd Zaini N.A., Daud N.‘A., Wan-Mohtar W.A.A.Q.I. (2025). Performance of mushrooms in fermented beverages: A narrative review. Beverages.

[11] Ribas-Agustí A., Martín-Belloso O., Soliva-Fortuny R., Elez-Martínez P. (2017). Food processing strategies to enhance phenolic compounds bioaccessibility and bioavailability in plant-based foods and bioavailability in plant-based foods. Crit. Rev. Food Sci. Nutr..

[12] Méndez-Galarraga M.P., Pirovani M.É., Vinderola G., Van de Velde F. (2025). Bioaccessibility of phenolic compounds in fermented strawberry-orange-apple-banana smoothies with Lactobacilli. Food Biosci..

[13] Markowiak P., Ślizewska K. (2017). Effects of probiotics, prebiotics, and synbiotics on human health. Nutrients.

[14] Raveschot C., Cudennec B., Coutte F., Flahaut C., Fremont M., Drider D., Dhulster P. (2018). Production of bioactive peptides by *Lactobacillus* species: From gene to application. Front. Microbiol..

[15] Marulo S., De Caro S., Nitride C., Di Renzo T., Di Stasio L., Ferranti P., Reale A., Mamone G. (2024). Bioactive peptides released by lactic acid bacteria fermented pistachio beverages. Food Biosci..

[16] Garzón A.G., Van de Velde F., Drago S.R. (2020). Gastrointestinal and colonic in vitro bioaccessibility of γ-aminobutiric acid (GABA) and phenolic compounds from novel fermented sorghum food. LWT.

[17] Alsharairi N.A. (2025). An analysis of three *Pistacia* species’ phenolic compounds and their potential anticancer and cytotoxic activities on cancer cells-a review. Curr. Issues Mol. Biol..

[18] Ripari Garrido J., Patrignani M., Puppo M.C., Salinas M.V. (2024). Nutritional and bioactive characterization of pistachio—A review with special focus on health. Explor. Foods Foodomics.

[19] Derbyshire E., Higgs J., Feeney M.J., Carughi A. (2023). Believe It or ‘Nut’: Why it is time to set the record straight on nut protein quality: Pistachio (*Pistacia vera* L.) focus. Nutrients.

[20] Fukui T.Y., Nakahara T.K. (2013). Method for Analyzing Oligomeric Proanthocyanidin (OPC). U.S. Patent.

[21] Wallace T.C., Giusti M.M. (2010). Evaluation of parameters that affect the 4-dimethylaminocinnamaldehyde assay for flavanols and proanthocyanidins. J. Food Sci..

[22] Van de Velde F., Pirovani M.E., Drago S.R. (2018). Bioaccessibility analysis of anthocyanins and ellagitannins from blackberry at simulated gastrointestinal and colonic levels. J. Food Compos. Anal..

[23] Aquino M.E., Drago S.R., Schierloh L.P., Cian R.E. (2025). Identification of bioaccessible glycosylated neuroprotective peptides from brewer’s spent yeast mannoproteins by in vitro and in silico studies. Food Res. Int..

[24] Re R., Pellegrini N., Proteggente A., Pannala A., Yang M., Rice-Evans C. (1999). Antioxidant activity applying an improved ABTS radical cation decolorization assay. Free Radic. Biol. Med..

[25] Benzie I.F.F., Strain J.J. (1996). The ferric reducing ability of plasma (FRAP) as a measure of “antioxidant power”: The FRAP assay. Anal. Biochem..

[26] Wang T.Y., Hsieh C.H., Hung C.C., Jao C.L., Lin P.Y., Hsieh Y.L., Hsu K.C. (2017). A study to evaluate the potential of an in silico approach for predicting dipeptidyl peptidase-iv inhibitory activity in vitro of protein hydrolysates. Food Chem..

[27] Donkor O.N., Stojanovska L., Ginn P., Ashton J., Vasiljevic T. (2012). Germinated grains—Sources of bioactive compounds. Food Chem..

[28] Hayakari M., Kondo Y., Izumi H. (1978). A rapid and simple spectrophotometric assay of angiotensin-converting enzyme. Anal. Biochem..

[29] Rauf A., Imran M., Abu-Izneid T., Iahtisham-Ul-Haq, Patel S., Pan X., Naz S., Sanches Silva A., Saeed F., Rasul Suleria H.A. (2019). Proanthocyanidins: A comprehensive review. Biomed. Pharmacother..

[30] Ribas L.E., Baravalle M.E., Gasser F.B., Renna M.S., Addona S., Ortega H.H., Savino G.H., Van de Velde F., Hein G.J. (2021). Extraction of phenolic compounds from the shells of pecan nuts with cytotoxic activity through apoptosis against the colon cancer cell line HT-29. J. Food Sci..

[31] Ky I., Le Floch A., Zeng L., Pechamat L., Jourdes M., Teissedre P.L. (2015). Tannins. Encyclopedia of Food and Health.

[32] Gentile C., Tesoriere L., Butera D., Fazzari M., Monastero M., Allegra M., Livrea M.A. (2007). Antioxidant activity of sicilian pistachio (*Pistacia Vera* L Var. Bronte) nut extract and its bioactive components. J. Agric. Food Chem..

[33] Mannino G., Gentile C., Maffei M.E. (2019). Chemical partitioning and DNA fingerprinting of some pistachio (*Pistacia vera* L.) varieties of different geographical origin. Phytochemistry.

[34] Noguera-Artiaga L., Salvador M.D., Fregapane G., Collado-González J., Wojdyło A., López-Lluch D., Carbonell-Barrachina Á.A. (2019). Functional and sensory properties of pistachio nuts as affected by cultivar. J. Sci. Food Agric..

[35] Zalazar-García D., Simirgiotis M.J., Gómez J., Tapia A., Fabani M.P. (2025). Andean *Pistacia vera* L. crops: Phytochemical update and influence of soil-growing elemental composition on nutritional properties of nuts. Horticulturae.

[36] Taghizadeh S.F., Davarynejad G., Asili J., Nemati S.H., Karimi G. (2018). Assessment of phenolic profile and antioxidant power of five pistachio (*Pistacia vera*) cultivars collected from four geographical regions of Iran. Avicenna J. Phytomed..

[37] Chirenje T., Chavez R., Rijal S., Arroyo I., Bañuelos G.S., Sommerhalter M. (2025). Phenolic composition and antioxidant capacity of pistachio seed coats at different tree ages under saline irrigation conditions. Agronomy.

[38] Munekata P.E.S., Domínguez R., Budaraju S., Roselló-Soto E., Barba F.J., Mallikarjunan K., Roohinejad S., Lorenzo J.M. (2020). Effect of innovative food processing technologies on the physicochemical and nutritional properties and quality of non-dairy plant-based beverages. Foods.

[39] Adebo O.A., Medina-Meza I.G. (2020). Impact of fermentation on the phenolic compounds and antioxidant activity of whole cereal grains: A mini review. Molecules.

[40] Méndez-Galarraga M.P., Pirovani M.E., García-Cayuela T., Van de Velde F. (2025). Fruit and vegetable beverages fermented with probiotic strains: Impact on the content, bioaccessibility, and bioavailability of phenolic compounds and the antioxidant capacity. Curr. Food Sci. Technol. Rep..

[41] Melini F., Melini V. (2021). Impact of fermentation on phenolic compounds and antioxidant capacity of quinoa. Fermentation.

[42] Plamada D., Vodnar D.C. (2022). Polyphenols—Gut microbiota interrelationship: A transition to a new generation of prebiotics. Nutrients.

[43] Liang Z., Huang Y., Zhang P., Fang Z. (2023). Impact of fermentation on the structure and antioxidant activity of selective phenolic compounds. Food Biosci..

[44] Morais S.G.G., da Silva Campelo Borges G., dos Santos Lima M., Martín-Belloso O., Magnani M. (2019). Effects of probiotics on the content and bioaccessibility of phenolic compounds in red pitaya pulp. Food Res. Int..

[45] Cuvas-Limon R.B., Ferreira-Santos P., Cruz M., Teixeira J.A., Belmares R., Nobre C. (2022). Effect of gastrointestinal digestion on the bioaccessibility of phenolic compounds and antioxidant activity of fermented aloe vera juices. Antioxidants.

[46] Serra A., MacIà A., Romero M.P., Reguant J., Ortega N., Motilva M.J. (2012). metabolic pathways of the colonic metabolism of flavonoids (flavonols, flavones and flavanones) and phenolic acids. Food Chem..

[47] Ter Z.Y., Chang L.S., Babji A.S., Zaini N.A.M., Fazry S., Sarbini S.R., Peterbauer C.K., Lim S.J. (2024). A review on proteolytic fermentation of dietary protein using lactic acid bacteria for the development of novel proteolytically fermented foods. Int. J. Food Sci. Technol..

[48] Zhang Q., Xing B., Sun M., Zhou B., Ren G., Qin P. (2020). Changes in bio-accessibility, polyphenol profile and antioxidants of quinoa and djulis sprouts during in vitro simulated gastrointestinal digestion. Food Sci. Nutr..

[49] Sunitha D. (2016). A review on antioxidant methods. Asian J. Pharm. Clin. Res..

[50] Noguera-Artiaga L., Pérez-López D., Burgos-Hernández A., Wojdyło A., Carbonell-Barrachina Á.A. (2018). Phenolic and triterpenoid composition and inhibition of α-amylase of pistachio kernels (*Pistacia vera* L.) as affected by rootstock and irrigation treatment. Food Chem..

[51] Swargiary A., Mritunjoy Kumar R., Mahmud S. (2023). Phenolic compounds as α-glucosidase inhibitors: A docking and molecular dynamics simulation study. J. Biomol. Struct. Dyn..

[52] Lalegani S., Ahmadi Gavlighi H., Azizi M.H., Amini Sarteshnizi R. (2018). Inhibitory activity of phenolic-rich pistachio green hull extract-enriched pasta on key type 2 diabetes relevant enzymes and glycemic index. Food Res. Int..

[53] Proencą C., Freitas M., Ribeiro D., Tomé S.M., Araújo A.N., Silva A.M.S., Fernandes P.A., Fernandes E. (2019). The dipeptidyl peptidase-4 inhibitory effect of flavonoids is hindered in protein rich environments. Food Funct..

[54] Singh A.K., Yadav D., Sharma N., Jin J.O. (2021). Dipeptidyl peptidase (Dpp)-iv inhibitors with antioxidant potential isolated from natural sources: A novel approach for the management of diabetes. Pharmaceuticals.

[55] Chen L., Wang L., Shu G., Li J. (2021). Antihypertensive potential of plant foods: Research progress and prospect of plant-derived angiotensin-converting enzyme inhibition compounds. J. Agric. Food Chem..

[56] Daskaya-Dikmen C., Yucetepe A., Karbancioglu-Guler F., Daskaya H., Ozcelik B. (2017). Angiotensin-I-Converting Enzyme (ACE)-inhibitory peptides from plants. Nutrients.

[57] Nongonierma A.B., FitzGerald R.J. (2019). Features of dipeptidyl peptidase IV (DPP-IV) inhibitory peptides from dietary proteins. J. Food Biochem..

[58] Daza-Rodríguez B., Martínez A.R., Padilla A.M., Lázaro J.M. (2023). Food-derived bioactive peptides with angiotensin-converting enzyme inhibiting effect: A systematic review. J. Pharmacol. Pharmacother..

