# Supplementary material for: Fermented Pistachio-Based Beverages as Novel Functional Foods: Improved Phenolic Bioaccessibility and Antidiabetic Properties
Source: Foods. 2026 May 8;15(10):1639. doi: 10.3390/foods15101639 (PMC13205561; doi:10.3390/foods15101639)
Supplement: Supplementary file 1 [file foods-15-01639-s001.zip › foods-4254824-supplementary.pdf]

Table S1. Phenolic compound content in intestinal digests from Argentine and Italian pistachio-based beverages.

**Phenolic Compounds (mg/100 g dw)**	**AC**	**APD4**	**APG3**	**IC**	**IPD4**	**IPG3**
Catechin	0.33 ± 0.01 [b]	0.12 ± 0.05 [a]	-	0.95 ± 0.01 [c]	0.14 ± 0.00 [a]	0.28 ± 0.02 [b]
Epicatechin	7.95 ± 0.16 [c]	6.70 ± 0.03 [c]	1.82 ± 0.20 [b]	7.12 ± 0.30 [c]	0.78 ± 0.00 [a]	0.72 ± 0.05 [a]
Rutin	0.02 ± 0.00 [a]	0.10 ± 0.02 [b]	0.19 ± 0.01 [c]	0.02 ± 0.00 [a]	0.17 ± 0.00 [c]	0.16 ± 0.02 [c]
Myricetin	-	-	-	0.01 ± 0.00	-	-
Quercetin	-	0.02 ± 0.00 [a]	0.08 ± 0.00 [c]	0.03 ± 0.00 [b]	0.09 ± 0.00 [c]	0.08 ± 0.00 [c]

Different lowercase letters within the same row indicate significant differences ($p \leq 0.05$).
